# Evaluation of the antioxidant and anti-inflammatory effect of sublingual glutathione on COPD patients

**DOI:** 10.25122/jml-2023-0161

**Published:** 2023-12

**Authors:** Ali Farag, Wassan Abass, Hyder Qassem

**Affiliations:** 1 Department of Clinical Pharmacy, College of Pharmacy, Mustansiriyah University, Baghdad, Iraq; 2 Department of Medicine, College of Medicine, Maysan University, Maysan, Iraq

**Keywords:** COPD, Glutathione, Interleukin-8, Glutathione peroxidase 1, Tumor necrosis factor-α

## Abstract

Glutathione (GSH) is a potent antioxidant and anti-inflammatory, proven effective in reducing treatment duration, prescribed doses, and hospitalization for several diseases. This study assessed the therapeutic response of chronic obstructive pulmonary disease (COPD) patients by measuring oxidative superoxide dismutase (SOD3), glutathione peroxidase 1 (GPX1), and inflammatory biomarkers such as tumor necrosis factor-alpha (TNF-α) and Interleukin-8 (IL-8) after sublingual administration of glutathione supplements. A cohort of 50 COPD individuals was involved and divided into two groups of 25 each. The first group received conventional therapy involving the administration of formoterol fumarate (12 µg inhaler) twice daily. The second group received the conventional treatment alongside sublingual glutathione (300 mg twice daily) for two months. The levels of serum IL-8, TNF-α, SOD3, and GPX1 were assessed before therapy, as well as at one and two months after treatment, in both cohorts. Both groups exhibited a notable reduction in the inflammatory mediators IL-8 and TNF-α when compared to their respective pre-treatment levels (P value <0.05). However, it is worth noting that the observed difference between the groups was not statistically significant (P value >0.05). The levels of SOD3 and GPX1 exhibited a substantial rise in both groups; however, they were found to be greater in group 2 compared to group 1 (P value >0.05). The administration of glutathione resulted in enhanced levels of antioxidant biomarkers among individuals diagnosed with COPD, accompanied by a minor and statistically insignificant decrease in the levels of the anti-inflammatory mediators IL-8 and TNF-alpha.

## INTRODUCTION

The Global Initiative for Chronic Obstructive Lung Disease (GOLD) defines chronic obstructive pulmonary disease (COPD) as a common treatable disorder characterized by significant airflow restriction caused by prolonged exposure to unpleasant particles and gases that alter the alveoli and airways [1]. It is a complicated disorder that results in disturbed pulmonary function, such as emphysema and chronic bronchitis [2, 3]. Based on the GOLD standard, the global prevalence of COPD in adults aged 30-79 years in 2019 was 10.3% [95% CI 8.2-12.8] or 391.9 million (95% CI 312.6-487.9) [3].

The rise in COPD is particularly evident in socioeconomically disadvantaged communities, where the prevalence of exposure to indoor air pollution, such as biomass smoke, is comparable to that of cigarette smoking as a contributing factor [4]. A growing body of research suggests that the disease is mostly influenced by heightened levels of oxidative stress within the pulmonary system, which may be attributed to many interrelated molecular mechanisms [5]. Reactive oxygen species (ROS) force endogenous antioxidant defenses to become compromised and overwhelming, leading to the development of oxidative stress [6]. Deterioration of the lung parenchyma (emphysema) and persistent inflammation and fibrosis of the small airways are hallmarks of COPD [7]. Given that COPD does not disappear when exposure to cigarette smoke is discontinued, it has been hypothesized that additional parameters, including autoimmunity or chronic infection, could also be responsible for disease progression [8]. There is evidence to show that oxidative stress plays a significant role in driving many pathogenic processes associated with COPD and its progression. This implies that the elevation of endogenous antioxidants or the use of exogenous antioxidants to mitigate oxidative stress may serve as a beneficial therapeutic approach. Nevertheless, the search for safe and effective antioxidants for COPD has proven to be a difficult task due to the elevated levels of oxidative stress in the pulmonary system [9]. The goal of treatment is to return the redox balance of the lungs to baseline while maintaining the advantages of oxidant transmission [10]. Numerous studies have demonstrated how oxidative stress affects lung function and causes antioxidant glutathione (GSH) to decline.

The GSH precursor, N-acetylcysteine (NAC), has been used in the treatment of many lung disorders, such as COPD and chronic bronchitis; previous studies revealed that the administration of high doses of medication has shown efficacy in the prevention of acute exacerbations. Additionally, consistent administration of dosages has been seen to effectively reduce the occurrence of flare-ups in individuals with chronic bronchitis [11]. Additionally, a meta-analysis revealed that those using NAC had considerably fewer flare-ups of COPD and chronic bronchitis [12]. NAC has shown encouraging outcomes when used as an additional therapy for idiopathic pulmonary fibrosis and is now included in the treatment guidelines for this condition [13]. In accordance with another study, supplementation with GSH successfully reduced the inflammatory response by stabilizing the cytokine imbalance in various viral illnesses [14].

The sublingual administration allows for direct absorption of glutathione via the buccal mucosa, bypassing the hepatic first-pass effect. Previous research findings have shown that the sublingual form of glutathione has superior bioavailability compared to oral administration [15, 16]

Using a sublingual formulation of GSH as a supplement might potentially serve as a beneficial approach to enhancing the inherent antioxidant defense mechanisms necessary for mitigating various acute and chronic illnesses. Nevertheless, the effectiveness of GSH therapy seems to be strongly correlated with the method of delivery, level of absorption, and the subsequent rise in its concentrations.

Hence, the objective of this study was to evaluate the treatment response by quantifying the concentrations of oxidative superoxide dismutase (SOD3), Glutathione Peroxidase 1 (GPX1), and inflammatory biomarkers such as Tumor Necrosis Factor Alpha (TNF-α), and Interleukin-8 (IL-8) after sublingual glutathione supplementation therapy.

## MATERIAL AND METHODS

### Study design

This prospective observational study was carried out between 1 June 2021 and 30 May 2022, at the Respiratory Disorders Unit of Al-Sadir Teaching Hospital in Misan governorate, Iraq, on patients with COPD admitted to the hospital for treatment.

### Sample size

**Figure 1:**
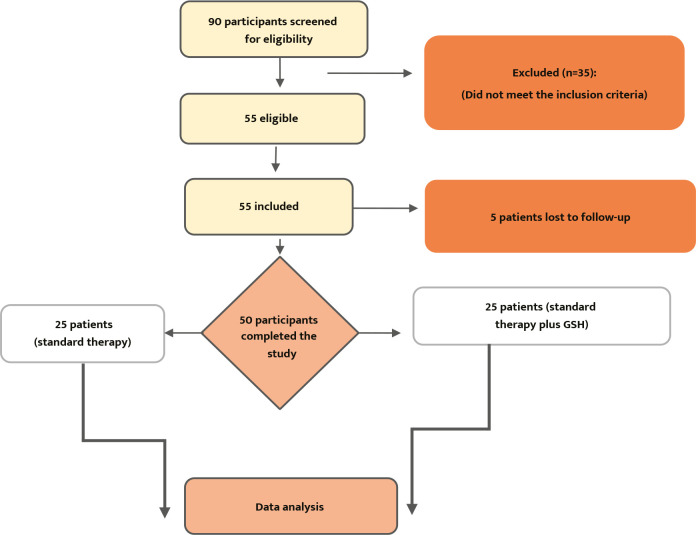
Flowchart of study participants

The sample size was estimated and computed using the computer program G*Power 3.1.9.7 (RRID: SCR 013726). The smallest total sample size was 42 patients, with an effect size of 0.50 and 95% power at a two-tailed alpha of 0.05 and a 95% confidence interval (f). Approximately 90 patients were screened for enrollment, but only 50 met the inclusion criteria, while the rest were excluded (Figure 1).

### Inclusion criteria

Patients with COPD, over 18 years of age, of both sexes, who could read and write in Arabic were included. COPD patients are diagnosed depending on the global initiative for COPD, namely the GOLD guidelines [17].

### Exclusion criteria

Patients diagnosed with asthma or other respiratory diseases except for COPD were excluded, as well as patients with COPD who were unable to perform acceptable spirometry. Patients on vitamins, antioxidant supplements, and corticosteroid therapy for other diseases were also excluded, as well as pregnant and breastfeeding patients and patients with diabetes mellitus, cardiovascular diseases, arthritis, and other systemic disorders.

### Study groups

The recruited patients were classified into two groups:

**Group 1** included 25 patients with COPD who received conventional therapy as stated by disease stage and severity for two months. The treatment consists of Formoterol fumarate (Recitor^®^; marketed by D&Fisher Co) 12 µg twice daily.

**Group 2** included 25 patients with COPD who received formoterol fumarate (Recitor^®^) 12 µg twice daily plus sublingual glutathione (Terry naturally Co-production, French Patent Number: FR2972327), 300 mg two times per day for two months.

### Equipment

Serum GPX, TNF-α, and IL-8 levels were estimated using the ELISA sandwich method. The kit supplied by Shanghai YL Biotech/China was used for measuring TNF-α (code: YLI0031) and IL-8 (code DE4700). Cusabio, China, provided SOD3 (code: CSB-EL022399HU), while GPX was supplied by Shanghai YL Biont (code: YLA1305HU). The principle of an assay is based on capturing the measured parameters between two antibodies followed by their quantification through an enzymatic reaction, yielding colored compounds to be tested at an optical density of 450 nm. The centrifuge was supplied by Eppendorf Corporate, the ELISA washer by Agilent BioTek, the incubator by Memmert, the plain tubes by Vacutest, the disposable syringes by Vacutest, and the autoanalyzer by Abbott.

### Clinical Evaluation

A pulmonologist interviewed all patients directly to assess illness signs, symptoms, medical history, and laboratory results.

### Bias

The authors took great precautions to guarantee that each qualified applicant had an equal chance of being chosen. In addition, the research was established for the settings considered, data completeness, and quality evaluation to ensure reduced bias during the sample selection and study technique by employing appropriate allocation sequence generation using a random number generator on a computer.

### Statistical analysis

To ascertain the impact of different variables on the research parameters, statistical analysis was performed using SPSS version 25 for Windows (RRID: SCR 016479). In this study, when data were reported as mean ± SD, the Kruskal-Wallis test was employed to compare means statistically, with statistical significance at P value <0.05. The paired t-test was used to compare pre- and post-treatment results. In contrast, the unpaired t-test was used to compare the variations in patient pre- and post-treatment outcomes between the two groups. The parameters were compared using one-way ANOVA with post hoc Tukey's test to identify which samples significantly differed from the other groups. In addition, the chi-square test was used to detect significant associations among demographic variables.

## RESULTS

Patient demographic characteristics are presented in Table 1. There were non-significant (P value <0.05) differences between groups regarding sex, age, and BMI.

**Table 1:** Demographic data

Characteristics		Study groups		P value
		Control (n=25)	Intervention (n=25)	
Gender	Male	19 (76%)	18 (72%)	0.76
	Female	6 (24%)	7 (28%)	
Age (years)	Mean ± SD	55.64±8.89	57.32±8.36	0.4
BMI (kg/m^2^)	Mean ± SD	28.76±4.04	28.4±3.61	0.7

**Figure 2:**
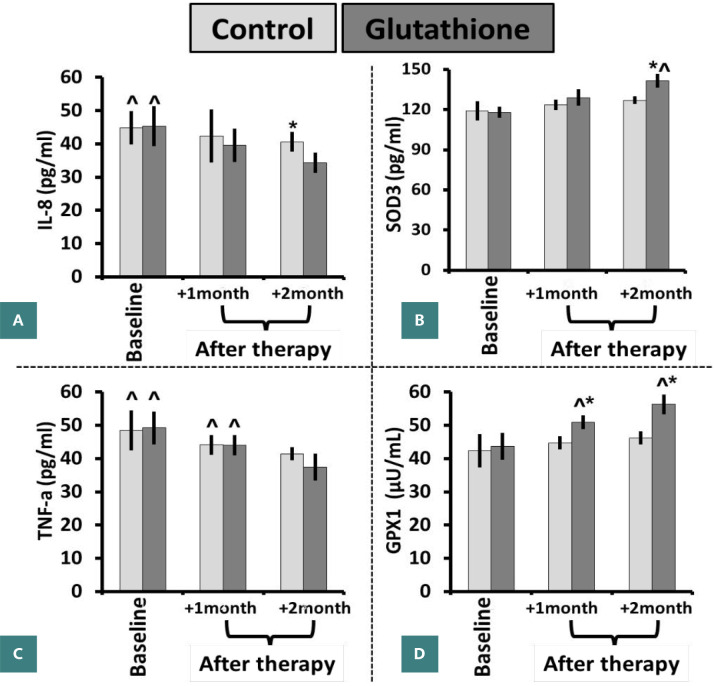
Glutathione modulated proinflammatory and antioxidant parameters at baseline and after therapy (1 and 2 months). (A) IL-8, (B) SOD3 (pg/ml), (C) TNF-α (pg/ml), and (D) GPX1 (µU/mL) levels. Data expressed as mean ± SD. *P value <0.05 compared to the control group. ^P value <0.05 at different time points in the same group.

The concentration of IL-8 in picograms per milliliter (pg/ml) exhibited a statistically significant decrease (P value <0.05) after two months in the control group. Specifically, the mean concentration decreased from 45 ± 5 pg/ml at baseline to 41 ± 3 pg/ml. Similarly, after a two-month course of glutathione treatment, the observed level (34 ± 3) exhibited a statistically significant reduction (P value <0.05) when compared to the first baseline measurement (45 ± 6). Nevertheless, a significantly greater decrease in levels was seen after a two-month administration of glutathione (34 ± 3) in comparison to the control group (41 ± 3) over the same duration (Figure 2A). The concentration of SOD3 (pg/ml) showed a statistically significant increase (P value<0.05) after two months of glutathione supplementation (142 ± 5), as compared to the first baseline measurement (118 ± 4) or the concentration seen after one month of intervention treatment (129 ± 6). In contrast, the experimental group exhibited no significant variations in SOD3 levels after one month (124 ± 4) or two months (127 ± 3) as compared to the control group (119 ± 7). Furthermore, a notable increase in the level was seen after a two-month administration of glutathione therapy (142 ± 5) in comparison to the control group (127 ± 3) (Figure 2B). The concentration of TNF-α (pg/ml) exhibited a statistically significant decrease (P value <0.05) after one month (44 ± 3) and two months (41 ± 2) in the control group, as compared to the baseline level (49 ± 6). Similarly, the level of glutathione considerably decreased (P value <0.05) after one month (44 ± 3) and two months (37 ± 4) of glutathione treatment, as compared to the baseline level (49 ± 5). Nevertheless, there were no notable disparities between the control and intervention cohorts at any time point (Figure 2C). The concentration of GPX1 (pg/ml) exhibited a statistically significant increase (P value <0.05) over two months in the glutathione group (56 ± 3), in comparison to the baseline measurement (44±4) and the concentration after one month of treatment (51 ± 2). In contrast, the experimental group exhibited no significant variations in SOD3 levels after one (45 ± 2) or two months (46 ± 2) compared to the control group (42 ± 5). Furthermore, a notable increase in the level of glutathione (56 ± 3) was seen after two months, in contrast to the control group (46 ± 2) (Figure 2D).

## DISCUSSION

After two months, the average IL8 concentration decreased significantly in both groups. However, there were no statistically significant distinctions in the reduction of IL8 between the groups. This suggests that glutathione and conventional therapy may have a similar therapeutic impact. The biomarker known as serum IL-8 has the potential to serve as a predictive tool in identifying individuals who exhibit heightened susceptibility to exacerbations of COPD. This is mostly due to the biomarker's sensitivity, ease of measurement, and cost-effectiveness. Zhang *et al*. identified IL-8 as a predictive factor for distinguishing patients with COPD who are prone to experiencing exacerbations [18]. Antonicelli *et al*. observed that thiol antioxidants, such as GSH, had inhibitory effects on neutrophil chemotaxis. This inhibition was attributed to the reduction in IL-8) production from macrophages and pulmonary epithelial cells [19].

Behr *et al*. revealed that the administration of high-dose NAC resulted in a slight elevation in intracellular glutathione levels. The observed augmentation is linked to a marginal reduction in oxidative activity rather than attributable to the fall in activation of bronchoalveolar cells [20]. Gosset *et al*. observed that the inclusion of GSH and NAC resulted in a significant reduction in the secretion of TNF-alpha (21.2% and 44.7% reduction, respectively), as well as IL-8. Conversely, the depletion of intracellular GSH through the use of buthionine sulphoximine led to a significant increase in the secretion of TNF-alpha and IL-8. Hence, NAC serves as a reliable synthetic precursor for intracellular GSH [21]. Sadowska *et al*. investigated the associations between indicators of oxidative stress and inflammation and observed significant alterations in these relationships after the administration of NAC therapy. Gaining insight into the fundamental processes that drive the inflammatory response in COPD) and its interconnectedness with oxidative stress has the potential to facilitate the development of preventative and therapeutic interventions aimed at arresting the progressive decline of lung function [22]. According to Rahman *et al*., under conditions of reduction, such as when intracellular GSH levels increase due to N-acetyl-L-cysteine treatment, NF-κB plays a regulatory role in controlling the expression of multiple genes associated with inflammation. The resulting products of these genes, including inducible nitric oxide synthase, proinflammatory NF-α, and IL-8, mediate inflammatory responses within the lungs [23].

The results of this study indicate a potential involvement of glutathione in activating this particular antioxidant enzyme. Statistically significant disparities in the levels of SOD3 were observed among the groups with COPD at one and two months. The variation in disease severity across patients and the potential inadequacy of conventional therapy in regulating oxidative levels at different phases of treatment may account for these findings. According to Yao *et al*., groups of extracellular SOD3 have a role in the pathophysiology of COPD by inducing the migration of inflammatory cells into the lungs afflicted by COPD. The drop in SOD3 levels is shown to be severe as the illness progresses. The experiments on murine lungs showcased that the presence of SOD3 restricts the development of emphysema and reduces the breakdown of the extracellular matrix due to oxidative stress in the murine lung. Consequently, the researchers concluded that the pharmacological activation of SOD3 within the pulmonary system might serve as a therapeutic intervention for managing COPD and emphysema [24]. The importance of SOD3 was also highlighted by Du *et al*., who looked for the association between SOD3 gene polymorphisms and the development of COPD genotype, and allele distributions of SOD3 was significantly different between the COPD and control groups [25].

Glutathione is found in higher concentrations in the fluid surrounding the epithelial lining compared to plasma, serving a vital protective function in the airspaces and epithelial cells by defending against and neutralizing oxidants in the extracellular environment. This endogenous antioxidant strategy is employed by the normal lung to mitigate the detrimental effects ROS. Enzymatic antioxidant defenses include SOD, GPX, and glutathione-S-transferase [26, 27]. Bentley *et al*. found that Gpx-1 has been shown to increase in patients with COPD initially. Furthermore, GPX activity is dramatically reduced in smokers and those with COPD, underlining its essential function in lung antioxidant defenses. Ongoing chronic inflammation may deplete the antioxidant defense system, leading to the loss of GPX [28]. Vibhuti *et al*. also demonstrate a reduction in plasma levels and activity of GPX in patients with COPD [29]. Vlahos *et al*. discovered that the antioxidant enzyme GPX-1 might be a possible therapeutic target for treating cigarette smoke-induced lung disease because of the increased oxidant burden in smokers and patients with COPD [30].

Cytokine involvement in the pathogenesis of the disease, specifically IL-8 and IL-6 [24], has been extensively studied for its role in tissue damage and subsequent disease progression [31]. However, the production of these cytokines occurs naturally inside the body via circulating immune cells or other cellular compartments that have important physiological activities. These cytokines play a role in the regulation of cellular and subcellular communication and maintain equilibrium with other anti-inflammatory cytokines, including IL-10, IL-4, IL-13, and TGF-β, reducing their potential harm [32, 33].

### Study limitations

This study did not assess the change in endogenous glutathione levels following sublingual glutathione delivery or quantify its half-life in circulation. Furthermore, this study did not investigate the advantages of increasing doses and a more extended treatment period, nor did it assess the genuine effect of different glutathione sublingual dosages on COPD.

There is a lack of research on the impact of sublingual glutathione on a broader range of antioxidant mediators (SOD1 and SOD2) or various oxidative stress indicators such as malondialdehyde and total antioxidant capacity. Detailed analysis of a variety of other anti-inflammatory biological markers, such as IL-6, IL-12, IL-17, IL-21, nuclear factor-kappa (NF-B), and interferon-gamma (IFN-), to gain a more integrated understanding of the impact of sublingual glutathione on other inflammatory mediators associated with COPD and whether it is capable of mitigating COPD exacerbating episodes [34-36].

Another limitation is the small sample size. A cohort study using sublingual glutathione adjuvant therapy with the treatment regimen would be beneficial in assessing glutathione involvement to avoid or minimize subsequent aggravating episodes that might require hospital admission in such individuals [37].

## CONCLUSION

Adding sublingual glutathione therapy to the treatment course of patients with COPD has dramatically reduced the levels of proinflammatory cytokines involved in the disease profile, such as IL-8 and TNFa. Concurrently, the antioxidant status was improved by measuring glutathione and superoxide dismutase enzymes. These findings suggest adding sublingual glutathione therapy to treating patients with COPD.

## Data Availability

Further data is available from the authors upon reasonable request.

## Special thanks

The authors wish to express gratitude to the Al-Sadir Teaching Hospital staff for their cooperation with this study and to all research volunteers.
